# Calpain signaling: from biology to therapeutic opportunities in neurodegenerative disorders

**DOI:** 10.3389/fvets.2023.1235163

**Published:** 2023-09-05

**Authors:** Elsayed Metwally, Hatim A. Al-Abbadi, Tarique Hussain, Ghulam Murtaza, Ahmed M. Abdellatif, Mahmoud F. Ahmed

**Affiliations:** ^1^Department of Cytology and Histology, Faculty of Veterinary Medicine, Suez Canal University, Ismailia, Egypt; ^2^Faculty of Medicine, University Hospital, King Abdulaziz University, Jeddah, Saudi Arabia; ^3^Animal Sciences Division, Nuclear Institute for Agriculture and Biology College (NIAB-C), Pakistan Institute of Engineering and Applied Sciences (PIEAS), Faisalabad, Pakistan; ^4^Department of Animal Reproduction, Faculty of Animal Husbandry and Veterinary Sciences, Sindh Agriculture University, Sindh, Pakistan; ^5^Department of Anatomy and Embryology, Faculty of Veterinary Medicine, Mansoura University, Mansoura, Egypt; ^6^Department of Surgery, Anesthesiology and Radiology, Faculty of Veterinary Medicine, Suez Canal University, Ismailia, Egypt

**Keywords:** calpain, calpainopathies, calpain inhibitor, calcium, neurodegeneration

## Abstract

Neurodegenerative disorders represent a major and growing healthcare challenge globally. Among the numerous molecular pathways implicated in their pathogenesis, calpain signaling has emerged as a crucial player in neuronal dysfunction and cell death. Calpain is a family of calcium-dependent cysteine proteases that is involved in many biological processes, such as signal transduction, cytoskeleton remodeling, and protein turnover. Dysregulation of calpain activation and activity has been associated with several neurodegenerative diseases, including Alzheimer’s, Parkinson’s, and Huntington’s diseases. Understanding the intricate structure of calpains is crucial for unraveling their roles in cellular physiology and their implications in pathology. In addition, the identification of diverse abnormalities in both humans and other animal models with deficiencies in calpain highlights the significant progress made in understanding calpain biology. In this comprehensive review, we delve into the recent roles attributed to calpains and provide an overview of the mechanisms that govern their activity during the progression of neurodegenerative diseases. The possibility of utilizing calpain inhibition as a potential therapeutic approach for treating neuronal dysfunctions in neurodegenerative disorders would be an area of interest in future calpain research.

## 1. Introduction

**Figure 1:**
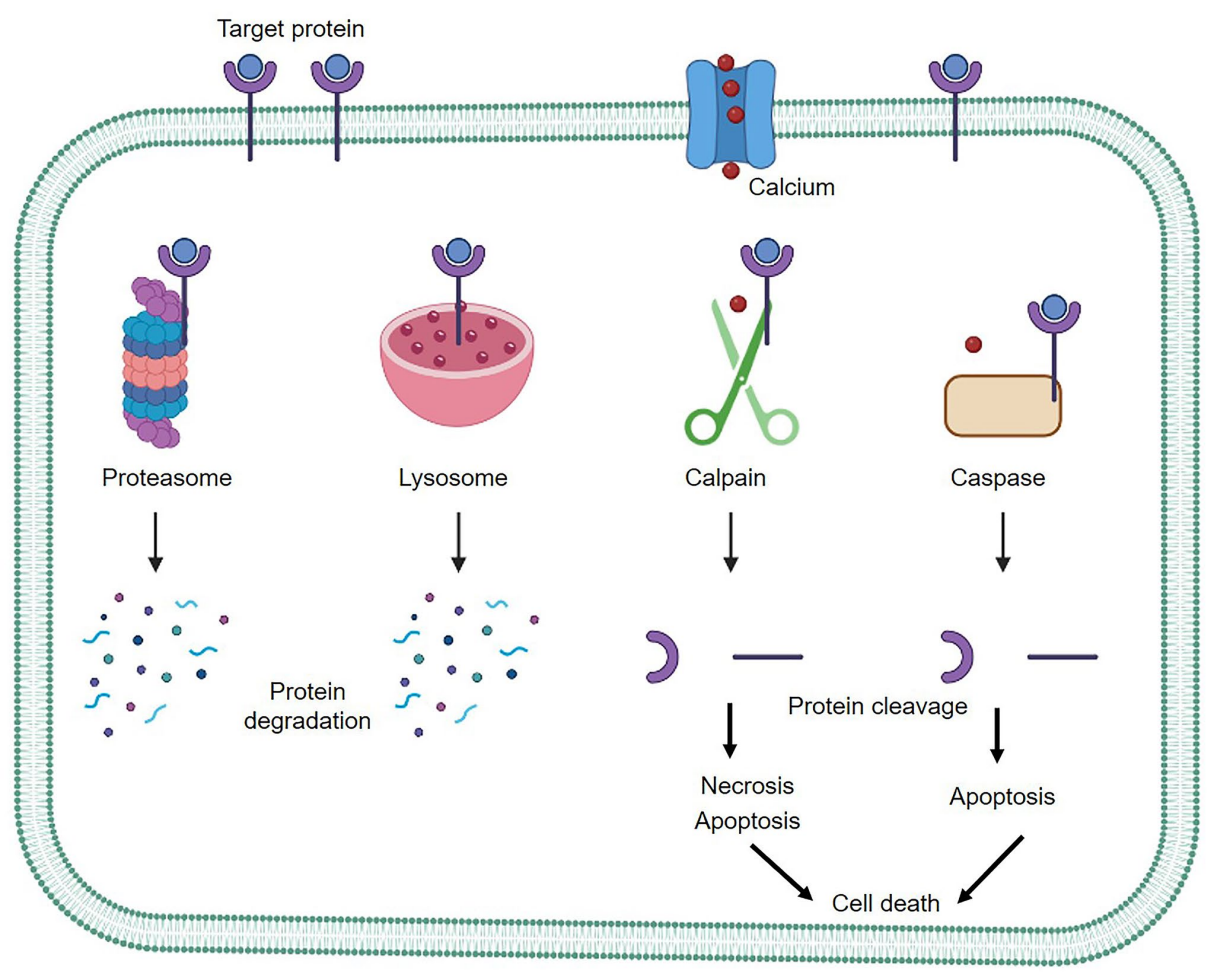
The three main protein degradation systems of mammalian cells. Proteasomes and lysosomes act as complete protein degradation pathways, while calpain functions in a calcium-dependent manner, cleaving target proteins to further regulate cellular activity.

Calpains are a class of intracellular cysteine proteases that are expressed in a wide range of tissues. They are activated by calcium and are functionally active at neutral pH (1, 2). Guroff originally identified calpains as calcium-dependent neutral proteases from rat brains (3). Although 15 calpain isoforms have been identified in the human genome, most of the studies have focused on two ubiquitous forms of calpain. μ-calpain (calpain-1) has a relatively high calcium binding affinity and is activated by micromolar calcium levels, while m-calpain (calpain-2) has a low calcium-binding affinity and is activated by millimolar calcium concentrations (4). There is a rich profile of biochemical and molecular data regarding the structural and biological properties of calpain (5, 6). Unlike most other proteases, such as proteasomes and lysosomes, which cause complete breakdown of their substrates upon activation, calpains cleave their substrates at limited and specific sites (7, 8). The ubiquitin-proteasome system is necessary for the breakdown and elimination of specific cellular components (9). The autophagy-lysosome system is another degradation system that completely breaks down non-specific cellular components (10). On the other hand, caspase is responsible for cleaving numerous cellular proteins, ultimately leading to cell death (11). Calpain and caspase are two distinct classes of proteolytic enzymes with different activation mechanisms and cleavage-site specificities. Calpain is involved in both necrotic and apoptotic cell death pathways, while caspase, especially caspase 3, is primarily associated with apoptosis, particularly in neuronal cells (12). Both proteases have critical roles in maintaining cellular homeostasis and regulating cell fate under different physiological and pathological conditions (Figure 1).

The activation of calpains is tightly regulated by intracellular calcium levels. Under normal physiological conditions, calpains remain in an inactive state due to the low concentration of calcium ions. However, when calcium levels increase in response to specific cellular signals or stressors, calpains are activated and initiate proteolytic cleavage of their target proteins. They regulate a diverse range of cellular functions when combined with the calpain small regulatory subunit 1 and the natural proteinaceous inhibitor calpastatin (13, 14). Calpain proteases are vital players in numerous physiological processes, including muscle function, neural development, cell signaling, and tissue remodeling. Their calcium-dependent activation and proteolytic activity make them key regulators of protein function and contribute to maintaining normal cellular and tissue homeostasis. Dysregulation of calpain activity has been implicated in several pathological conditions, including neurodegenerative diseases as well as other systematic disorders (15, 16). Neurodegenerative disorders, such as Alzheimer’s disease (AD), Parkinson’s disease (PD), and Huntington’s disease (HD), are characterized by progressive and irreversible loss of neurons, leading to cognitive decline, motor impairment, and behavioral changes. Despite extensive research, effective disease-modifying treatments remain elusive. Calpain signaling, with its diverse roles in cellular processes, has attracted attention as a potential therapeutic target in these neurodegenerative diseases.

This review aims to comprehensively cover the present research and research perspectives concerning calpain activation mechanisms. Furthermore, it sheds light on the role of calpain in neurodegenerative diseases and discusses a prospective therapeutic approach to using the calpain system as a novel therapeutic tool in neurodegenerative disorders.

## 2. Calpain structure and activation mechanisms

**Figure 2:**
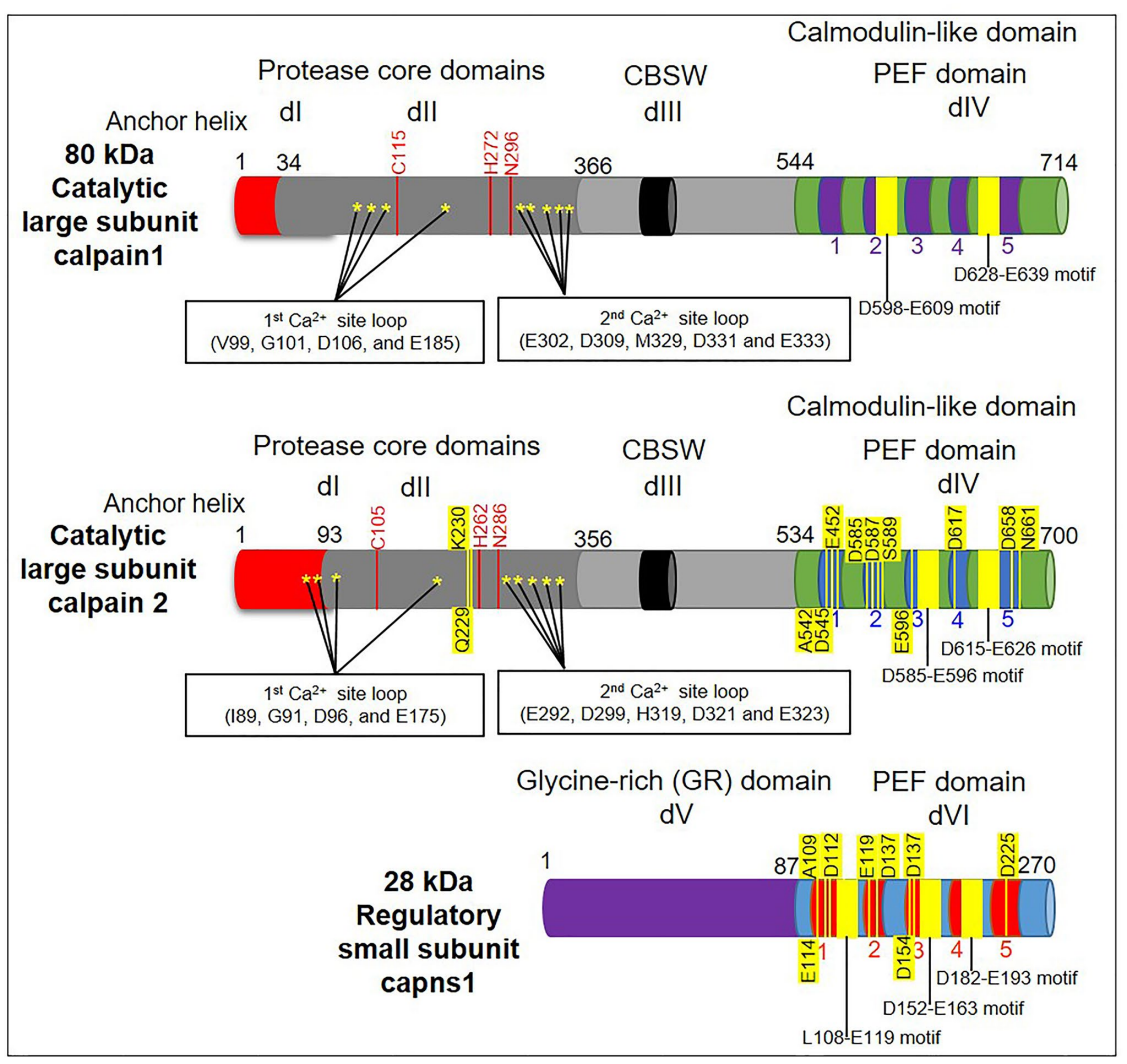
A schematic diagram depicting the domain structure of calpains’ subunits (large and small). The diagram highlights the presence of calcium-binding sites (denoted by stars) in domains I and II (dI and dII/protease core domains). Note that dII contains the active sites responsible for the catalytic activity (red lines). Meanwhile, the dIII/calpain β sandwich domain (CBSW) is a flexible linker between protease core domains and the penta-EF-hand domain [PEF(L)/dIV]. Conventional calpains generally consist of a large catalytic subunit that is composed of four domains (dI–dIV), as well as a small regulatory subunit that is composed of two domains: the glycine-rich domain (GR/dV) and the penta-EF-hand domain [PEF(S)/dVI]. Both subunits have a C-terminal calmodulin-like domain with a penta-EF-hand motif and calcium binding sites (yellow lines).

It’s worth noting that the structure and arrangement of calpain domains can differ across different isoforms and species. The information provided here represents a general overview of the domains found in calpain proteins but may not cover all possible variations. Structurally, calpain is formed from a large catalytic subunit with four domains: protease core domains (PC1 and PC2 or dI and dII), calpain β sandwich domain (CBSW or dIII), and penta-EF-hand domain [PEF(L) or dIV], in addition to a small regulatory subunit with two domains: glycine-rich domain (GR or dV) and penta-EF-hand domain [PEF(S) or dVI] (6, 17, 18). The N-terminal dI serves as calpain’s primary regulatory region. It contains a calcium-binding EF-hand motif responsible for sensing intracellular calcium levels. In the absence of calcium, dI interacts with dIII, maintaining the calpain in an inactive conformation. The interaction of the regulatory and catalytic subunits is facilitated by dII, which keeps the enzyme autoinhibited in the absence of calcium. The large dIII harbors the active site cysteine residue responsible for proteolytic activity. In the inactive state, it forms a protective interaction with dI and remains inaccessible. The small dIV is involved in the structural stability of the calpain enzyme (6) (Figure 2).

The presence of calcium-binding PEF-hand motifs in domains IV and VI of calpains is the main mechanism mediating calcium-dependent activation of calpains (19). The 3D structure of calpain (18) demonstrates that the PEF-hand domain is located close to the protease domain. The structural modification upon calcium binding to the PEF-hands mediates aligning the active catalytic sites of calpain and, in turn, leads to further activation (20). Additionally, biochemical investigations revealed that the non-PEF-hand domains and dII particularly acted as calcium switches to align the catalytic sites at dII (21). Importantly, Davies and colleagues pointed out a paired new calcium-binding site within the crystal structure of μ-calpain’s dII (20). It is known that these sites have a strong affinity for calcium and are necessary for complete enzymatic activity together with domains III, IV, and VI (22) (Figure 2).

**Figure 3:**
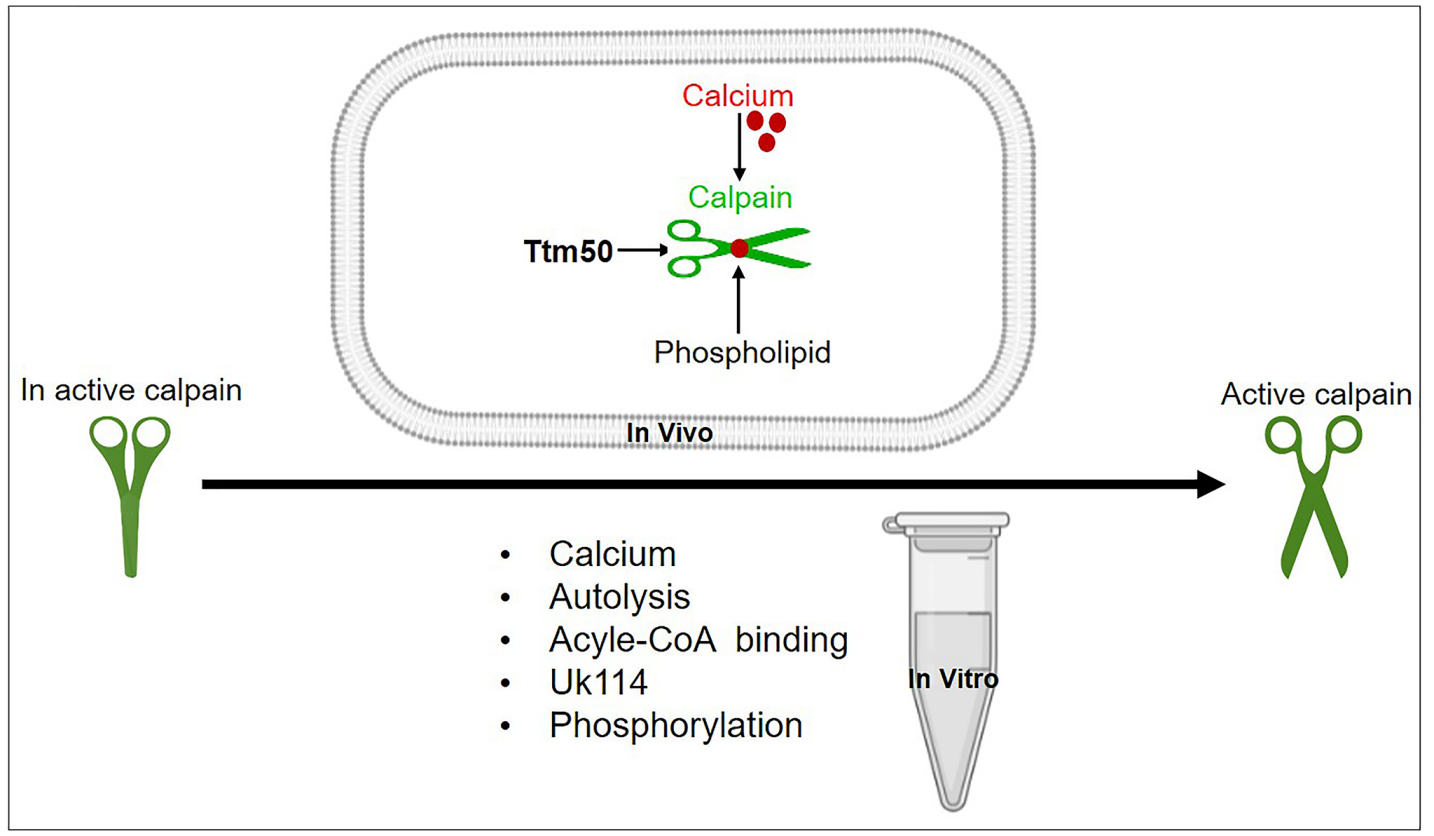
A schematic representation of different mechanisms proposed for calpain activation *in vitro* and *in vivo*. Calcium, autolysis, acyle-CoA-binding, UK114, phospholipids, phosphorylation, and other common activators for various enzymes activate conventional calpains; hence, they could not be employed as a specific activator. Although these activators have been identified, none have been validated *in vivo*. Only Ttm50 and phospholipids were proven to effectively activate calpain *in vivo*.

Calpain dIII has eight strands that are comparable to the C2 domain. Calcium is known to interact with the latter domain. In addition, an acidic loop inside calpain dIII has been postulated to bind calcium and promote calpain activity (23). Whereas the calcium-binding activity of domains IV and VI is well characterized. The discovery of two unanticipated calcium-binding sites in the structure of calpain dII led to the identification of a unique mechanism behind calpain’s calcium-dependent activation (20). The discovery of all calcium-binding sites within multiple domains (dII, dIV, and dVI) raises the question of how calcium binding to all three domains regulates calpain activation. The process of calpain activation includes the sequential rather than simultaneous activation of these domains. So, calcium activation of calpain in two steps has been proposed (20). As shown in Figure 2, the first step is the conformational shift in dIV and dVI caused by calcium binding, which disrupted the connection between the N-terminal α-helix of dI and the PEF-hand motif of dVI, promoting conformational change and separation of the small subunits from the large ones. The second step is the realignment of the active site cleft created by calcium binding to dII (24). The above-mentioned mechanism of calpain activation by dII binding to calcium is greatly conserved in calpains from *Drosophila* to humans. Furthermore, calpain members have varied calcium requirements. Moreover, non-classical calpains are calcium-independent. The binding of calcium ions is primarily responsible for controlling calpain activation. The activation process involves several steps and can vary depending on the specific isoform and cellular context. The general mechanisms of calpain activation are as follows: calpain is activated in two stages. First, a small amount of calcium binds to the calpain regulatory subunit, causing a conformational shift and exposing the active site of the enzyme. However, this low-affinity calcium binding is inadequate for full activation. The second stage is to raise the calcium concentration, which binds to the enzyme’s catalytic subunit and causes complete activation. Previous studies have shown that dI of calpain has a short N-terminus that undergoes autolytic cleavage either during its pre-or post-activation (25, 26). The autolyzed fragments showed reduced molecular weights after calcium addition (27). It was observed that protein–protein interactions change calcium sensitivity. As shown in Figure 3, possible candidate proteins such as UK114, acyl-CoA, and others co-purified with calpain act by increasing calpain autolysis *in vitro* (28, 29). Unfortunately, the *in vivo* activation of calpain by these activators has not been investigated further, and the mechanism by which calpain is regulated remains unclear. Epidermal growth factors have been shown to activate ERK/MAP kinase downstream of m-calpain (26). Calpain is also activated by ERK and PKA-mediated phosphorylation and deactivated by PP2A-mediated dephosphorylation. It is still controversial whether ERK-induced calpain phosphorylation still needs calcium for activation. However, it has been suggested that such phosphorylation leads to calpain binding to PP2B (30). Furthermore, brain-derived neurotrophic factor (BDNF), a key molecule required for synaptic plasticity and long-term potentiation, is reported to activate m-calpain through ERK-mediated phosphorylation at serine 50 (31). Collectively, the discovered processes contribute to the cellular activity of calpain, adding complexity to calpain activity regulation. Recently, it was found that tiny tim50 (Ttm50), a *Drosophila* homolog of the human tim50, a subunit of the TIM23 complex required for protein transport across the mitochondrial inner membrane, interacts with calpain and facilitates its activation both *in vitro* and *in vivo* (Figure 3), providing a novel mechanism for regulating calpain activity (16, 32). This Ttm50-calpain interaction puts the calpain in high proximity to calcium, which in turn facilitates calpain activation in the cell. Previous studies have demonstrated that variable intracellular localization, particularly membrane targeting in the presence of calcium (33, 34), regulates calpain activity. PIP2 was previously reported to be essential for m-calpain activation and to function as a cofactor for the enzyme by promoting its anchoring to the plasma membrane (35). Calpain molecules’ associations with the cytoplasmic surfaces of subcellular organelle membranes have been extensively investigated (36). The Golgi/ER calcium stores contain most of the localized calpain (32). Ttm50 also mediates calpain activation by controlling the localization of calpain at the Golgi/ER (16, 32, 37). Interestingly, not only is the localization of calpain in the Golgi calcium-dependent, but calcium also strengthens the interaction between Ttm50 and calpain (32). Therefore, calcium-dependent calpain localization at the Golgi/ER via Ttm50 may explain calpain activation *in vivo* (Figure 3). Moreover, Ttm50 protein binding not only anchors calpain in the Golgi/ER calcium stores, but it also increases calpain’s sensitivity to calcium (32). It’s important to note that the subcellular localization of calpain activation can vary depending on the cell type and the specific signaling context. Calpains are found in various subcellular compartments, including the cytoplasm, nucleus, mitochondria, and plasma membrane (38). Their subcellular localization determines the substrates they can access and their functional effects within the cell.

## 3. Calpain in neuronal development

During neuronal development, calpains participate in various processes such as neurite outgrowth, axon guidance, synaptogenesis, and dendritic spine formation. Calpain-mediated proteolysis is required for the dynamic rearrangement of the cytoskeleton, which is essential for neurite extension and growth cone guidance (39). By cleaving cytoskeletal proteins, calpains modulate the stability and plasticity of neuronal structures. Calpains play a multifaceted role in neuronal development, exerting regulatory control over neurogenesis, synaptogenesis, and activity-dependent plasticity. Through their proteolytic activity, calpains modulate key cellular processes involved in the formation, maturation, and refinement of neuronal circuits (39). Dysregulation of calpain activity during neuronal development is associated with neurodevelopmental disorders, highlighting the significance of calpain-mediated processes in maintaining proper brain development and function. The influx of calcium is increased in multiple processes, including neuronal excitability, stress, and muscular contraction (40, 41). Based on genetic and optogenetic control of calcium influx at sensory neurons (42) and neuromuscular synapses (43), calpains act downstream of calcium influx during normal development. An intrinsic neuronal characteristic that increases local calcium transits causes the activation of calpain. In fact, localized alterations in the excitability of the dendritic branch are observed across multiple mammalian neuron types (44).

**Figure 4:**
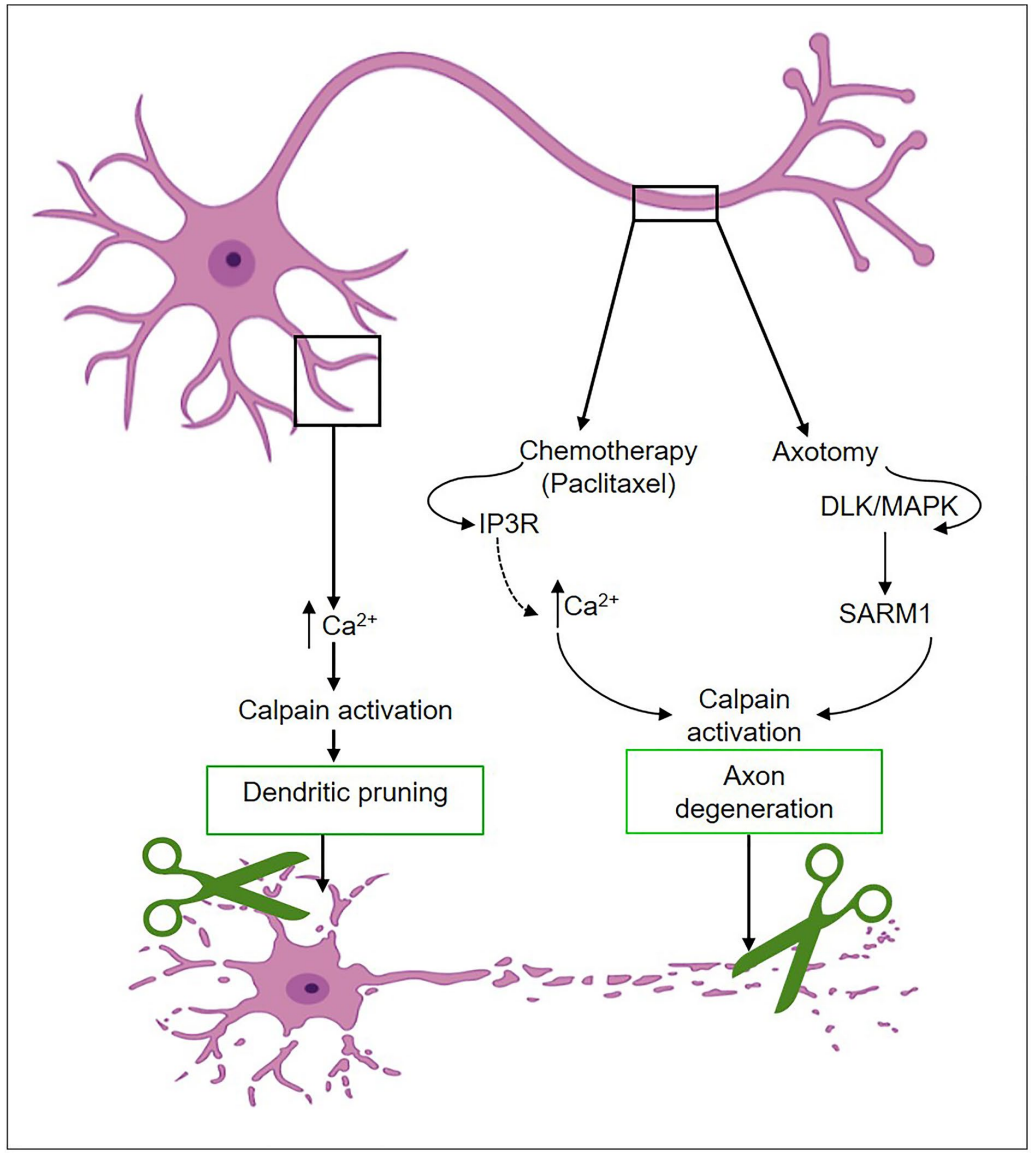
Calpain activation mechanisms during dendritic remodeling and axon degeneration.

Axon and dendritic pruning of neural connections facilitate the development of strong, mature circuits. There have been several reports on signaling pathways that are critical for axon and dendritic pruning and refinement (45–47). The ubiquitin-proteasome system, caspases, and calpains are triggered during development upon the initiation of self-destruction mechanisms in the dendrites and axons (46–48). These pathways exhibit either parallel or intersecting behavior. Upon activation of caspase, which results in the depletion of calpastatin, calpains with a higher degree of substrate specificity induce the proteolysis of cellular proteins such as neurofilaments, leading to axonal degeneration (49, 50). Highly branching dendritic arbors are selectively pruned during brain development to establish optimal connections. Kanamori et al. (42) reported that transient calcium waves are localized to particular dendrites before pruning the sensory neurons. By using a fluorescent calcium indicator, they visualized shifts in calcium concentration in intact *Drosophila* and detected calcium transients in groups of dendrites before dendrite severing and fragmentation but not in axons or cell bodies (42) (Figure 4). Calcium waves were not coordinated across dendritic neuron classes, and dendrites were pruned in the same temporal order that calcium transients emerged. Dendrites with the earliest calcium transients were pruned first, then those that initiated calcium waves. Voltage-gated calcium channels (VGCCs), the main calcium regulator before pruning (42), control the development of calcium transients at the same time as increasing the excitability of dendrites. Calpain is essential for dendritic pruning. Loss of function in genes encoding a subunit of VGCC and calpain-A or B, in particular, causes improper pruning in sensory neurons (42). Furthermore, calpain enhances the downregulation of GluRIIA at *Drosophila* neuromuscular synapses throughout development in response to calcium influx via calcium channels or from intracellular storage sites such as the Golgi and ER (43). It has been demonstrated via genetic and optogenetic studies that calpains activated by calcium are likely to occur in particular physiological situations at neuromuscular synapses (43). In the zebrafish spinal cord, high-amplitude, long-duration calcium transients elicit myelin sheath retraction, which is associated with calpain activity. Because of calpain activation, this mechanism most likely includes the localized destruction of cytoskeletal components (51).

Neurogenesis occurs primarily during embryonic development and persists in specific subdivisions of the adult brain, such as the cerebellum, hippocampus, and olfactory bulb (52). Calpains play a crucial role in regulating neurogenesis by modulating multiple steps involved in neuronal differentiation and maturation. Studies have revealed that calpain activity regulates neuronal migration, dendritic arborization, and the dynamics of axonal growth cones (53, 54). Calpains also affect the balance between neuronal survival and apoptosis, ensuring appropriate neuronal development and integration into functional circuits (39). Synaptogenesis is a fundamental process in establishing neuronal connectivity and neural circuitry (55). Calpains regulate synaptogenesis by influencing the dynamic remodeling of the cytoskeleton and synaptic proteins. Calpain-mediated proteolysis of cytoskeletal proteins, such as spectrin and microtubule-associated proteins, contributes to the morphological changes necessary for synapse formation and maturation. Furthermore, calpains modulate the turnover and localization of synaptic proteins, including receptors and scaffolding molecules, influencing synaptic plasticity and neuronal connectivity (56). During cerebellar development, the dendritic arborization of Purkinje cells and other cerebellar neurons is orchestrated by calpain-mediated cytoskeletal remodeling, which affects the shape and complexity of dendritic trees (57). Overall, calpain in the cerebellum plays a vital role in shaping the development of this brain region. Its precise functions vary depending on the specific developmental stage and cell types involved. Proper regulation of calpain activity is crucial for establishing a well-organized and functional cerebellum, and its dysregulation could lead to neurological disorders.

Calpains play a significant role in activity-dependent plasticity, which refers to the ability of neurons to modify their synaptic strength in response to neuronal activity patterns (58). Calcium influx, triggered by neuronal activity, activates calpains, leading to the proteolysis of key synaptic proteins. This process allows for the dynamic remodeling of synapses and the fine-tuning of neuronal connections based on experience and environmental cues. Calpain activity in activity-dependent plasticity is crucial for synaptic maturation, refinement, and functional integration within neuronal circuits (58). Dysregulation of calpain activity during neuronal development has been implicated in various neurodevelopmental disorders. For instance, abnormal calpain activation has been observed in autism spectrum disorders (59). It contributes to alterations in dendritic spine morphology and synaptic protein expression. Calpain dysregulation has also been linked to intellectual disability, schizophrenia, and other neurodevelopmental conditions (60). Understanding the precise mechanisms underlying calpain dysfunction in these disorders may provide valuable insights into their pathogenesis and potential therapeutic targets.

## 4. Calpain in neuronal injury

Neuronal injury is a complex process involving various molecular events that can lead to degeneration and loss of neuronal function. Axon degeneration is a self-destruction process that occurs during development, injury, and disease to eliminate damaged or unnecessary axons. Recent research has yielded interesting discoveries about the key role of calpain in Wallerian-type axon degeneration (61, 62). Calcium regulation disruptions play a critical role in establishing the activation of calpain proteases, hence contributing to axon degeneration upon toxic or traumatic damage (63, 64). Calpain dysregulation has been linked to aberrant proteolysis of numerous neuronal proteins, which may have a detrimental effect on axonal integrity, synapse function, and overall neuronal survival. Although it is believed that an increase in axonal levels of calcium is required for calpain activation, which leads to axon degeneration and elimination, the source of this calcium increase is still unclear (50). Many investigations have revealed that the release of calcium from intracellular stores causes calpain activation and subsequent axon degeneration after chemical or mechanical injury (64, 65). The administration of paclitaxel, a widely utilized chemotherapeutic medication for breast, ovarian, lung, and other cancer patients, leads to the development of peripheral neuropathy (66). Paclitaxel has been found to activate calpain proteases through alterations in IP3R1 phosphorylation and intracellular calcium influx (66). At the same time, paclitaxel selectively lowers the expression of the Bcl2 family member Bclw (IP3R1 inhibitory binding partner) in axons (Figure 4). This increases the levels of cytosolic calcium, which activates calpain (66). In addition to the Bclw-IP3R1 pathway, other pathways have been proposed to be involved in calpain-mediated axonal degeneration through calcium modulation or disruption of intracellular calcium homeostasis. These pathways encompass the direct influx of calcium through the damaged plasma membrane, activation of VGCC in the plasma membrane, activation of RyR and IP3R, and inhibition of SERCA in the ER/Golgi. One or more of these pathways may contribute significantly, depending on the type of neurons and the specific degenerative insult.

The signaling pathway involving sterile alpha and TIR motif-containing 1 (SARM1) and the toll-interleukin-1 receptor (TIR) are other mechanisms that lead to calpain activation in axotomy-induced degeneration (67), as shown in Figure 4. The lack of the *Drosophila* homolog of SARM1 (dSarm) inhibits Wallerian degeneration in *Drosophila* for an extended period after axotomy (68). Meanwhile, axons severed from SARM1-null mutant mice showed improved long-term survival *in vitro* and *in vivo*. These results imply that SARM1-mediated pro-degenerative signaling is conserved in mammals (68). Furthermore, SARM1 is necessary for the initiation of a localized destructive process involving the rapid breakdown of nicotinamide adenine dinucleotide (NAD^+^) following damage. The SARM1 TIR dimer activation increases its NAD^+^ hydrolase activity, resulting in the swift breakdown of NAD^+^. Surprisingly, enhanced NAD^+^ synthesis may reverse the axon damage triggered by SARM1 (69). Activated SARM1 causes rapid activation of mitogen-activated protein kinase (MAPK) (67). Endogenous SARM1 has also been shown to be necessary for MAPK activation after nerve injury in both *in vivo* and *ex vivo* cultures of retinal ganglion cells. These results support the presence of a pro-degenerative SARM1-MAPK pathway (67, 70) (Figure 4). On the other hand, high levels of axonal calpastatin reduced injury-induced degeneration in cultured neurons (71). This finding is consistent with the demonstrated protective effect of calpastatin in mice after its overexpression (72). Calpain action is dependent on high calcium levels, and the depletion of endogenous calpastatin alone is insufficient to induce axon degeneration *in vitro* and *in vivo* (50, 66). Understanding the intricate pathways by which calpain contributes to neuronal damage might lead to the development of innovative therapies that target neurodegenerative disorders and increase neuronal survival. Ongoing research in this field will be critical in unraveling the complex nature of calpain-mediated neuronal injury as well as discovering effective therapeutic strategies.

## 5. Calpain in spinal cord injury

**Figure 5:**
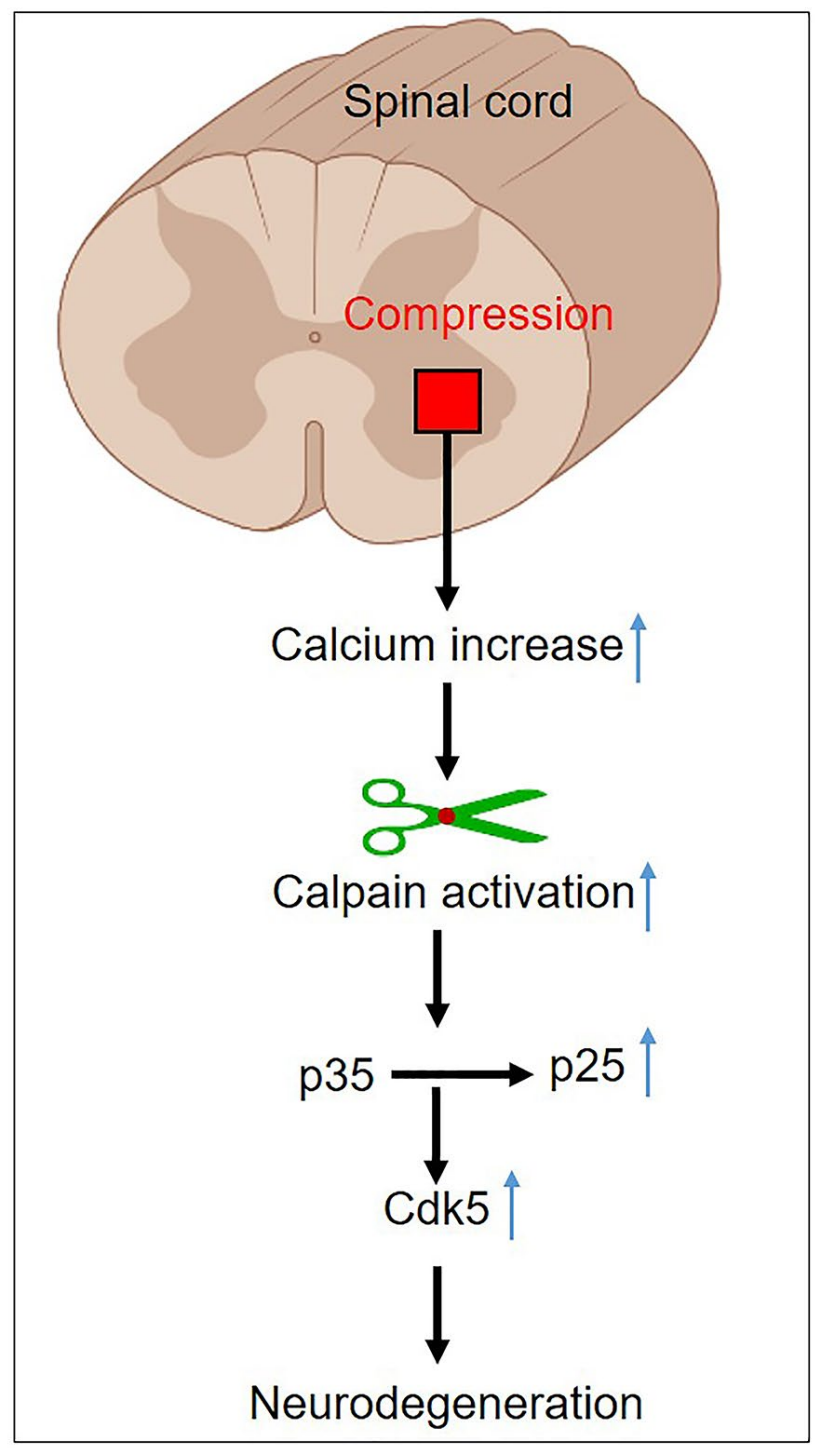
Mechanism of calpain activation in spinal cord injury.

Spinal cord injury (SCI) is a devastating condition characterized by the disruption of neural connections, resulting in sensory and motor dysfunction. The abrupt deprivation of mobility following an SCI brings about profound life-altering consequences (73), compelling researchers to focus their efforts on exploring innovative therapeutic approaches (74, 75). SCI occurs due to initial mechanical trauma, leading to physical and biochemical alteration. It interferes with the neural communication between the brain and the body, giving rise to paralysis or paraparesis (76). SCI is often accompanied by the formation of a hematoma as well as oxidative and inflammatory responses (77). Integrated therapy regimens have yielded partially successful outcomes in the treatment of acute SCI (78). When an SCI occurs, it triggers a cascade of events that can lead to secondary damage, exacerbating the initial injury. One of these events is the activation of calpain. Calpain activation in SCI can occur due to the influx of calcium ions into cells, which is a consequence of the disruption of the blood-spinal cord barrier and the release of excitatory neurotransmitters (Figure 5).

Calpain activation is a well-known component of neurodegeneration, causing damage to the brain and spinal cord in both human and animal models (16, 22). Calpain activation plays a pivotal role in the secondary injury cascade following SCI, contributing to neuronal death and functional impairment. Targeting calpain with inhibitors holds significant promise as a therapeutic strategy for mitigating the detrimental effects of SCI. Calpain activation induces the cleavage of p35 into p25, resulting in prolonged activation and the misplacement of cyclin-dependent kinase 5 (cdk5), leading to neuronal death (Figure 5). Furthermore, cdk5 dysregulation hyperphosphorylates tau, further exacerbating neuronal death (79, 80). As a result, using calpain inhibitors appears to be a potential therapy option for acute SCI. In an adult dog SCI model, blocking calpain activity with a particular calpain inhibitor (PD150606) combined with methylprednisolone sodium succinate (MPSS) reduced neuronal death, enhanced the canine Basso, Beattie, and Bresnahan locomotor score, and improved neuroprotection (81). These experimental findings demonstrated significantly lower neuronal loss and microglial cell invasion. The combination therapy substantially prevented calcium-induced p35 cleavage, CDK5 activation, and tau phosphorylation. These findings imply that early PD150606 treatment after severe SCI may prevent future neurodegeneration by inhibiting calpain. Plantier and his colleagues revealed that calpain activation is the upstream mechanism of motoneurons hyperexcitability after SCI in a rat model. Calpain-mediated voltage-gated sodium channel and potassium/chloride extruder KCC2 cleavage, respectively, increase persistent sodium current and inhibit lumbar motoneurons. Calpain inhibition reduces spasticity via normalizing persistent sodium current and the intensity of inhibitory transmission. These results shed light on a unique cellular process that contributes to spasticity and suggest a possible therapeutic approach. Calpain inhibition seems to be a viable therapeutic option rather than targeting voltage-gated sodium channels or KCC2 independently since it impacts two pathways simultaneously (82). These results have fueled interest in calpain inhibitors as potential therapeutic medications for acute SCI. Continued research efforts and clinical trials are essential to fully understanding the therapeutic potential of calpain inhibitors and translating these findings into effective treatments for SCI patients, ultimately improving their quality of life and functional outcomes. The role of calpain in SCI has been extensively studied, and there is growing interest in developing therapeutic strategies to target calpain activity and mitigate its detrimental effects. Several experimental approaches have been explored, including the use of calpain inhibitors and genetic manipulation to modulate calpain expression or activity. These studies have shown promising results in reducing secondary damage and promoting neuroprotection in preclinical models of SCI.

## 6. Neurodegenerative disorders involving calpain activation

**Figure 6:**
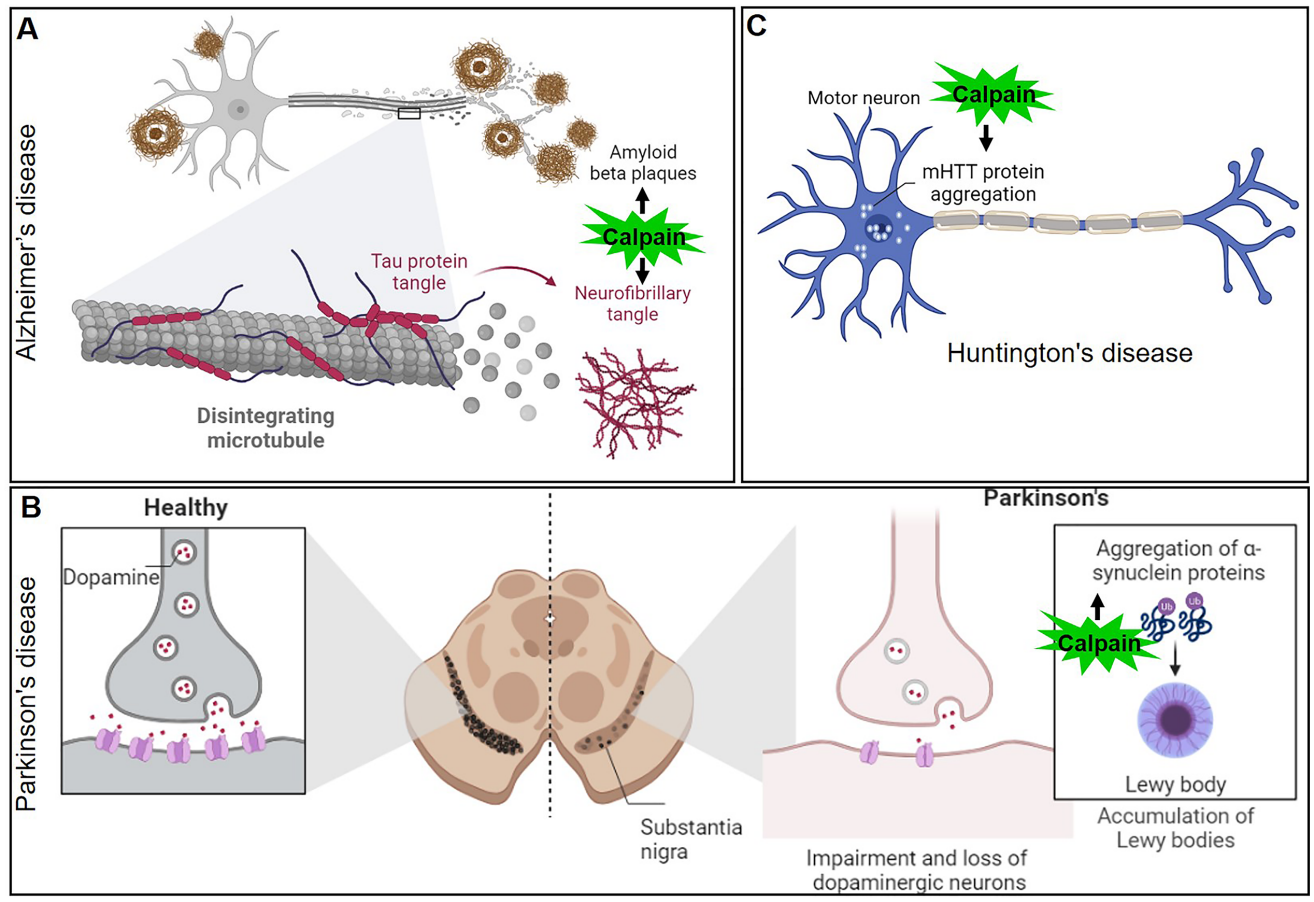
Mechanisms of calpain activation in the pathogenesis of various neurodegenerative disorders **(A)** Alzheimer’s disease, **(B)** Parkinson’s disease, and **(C)** Huntington’s disease.

**Figure 7:**
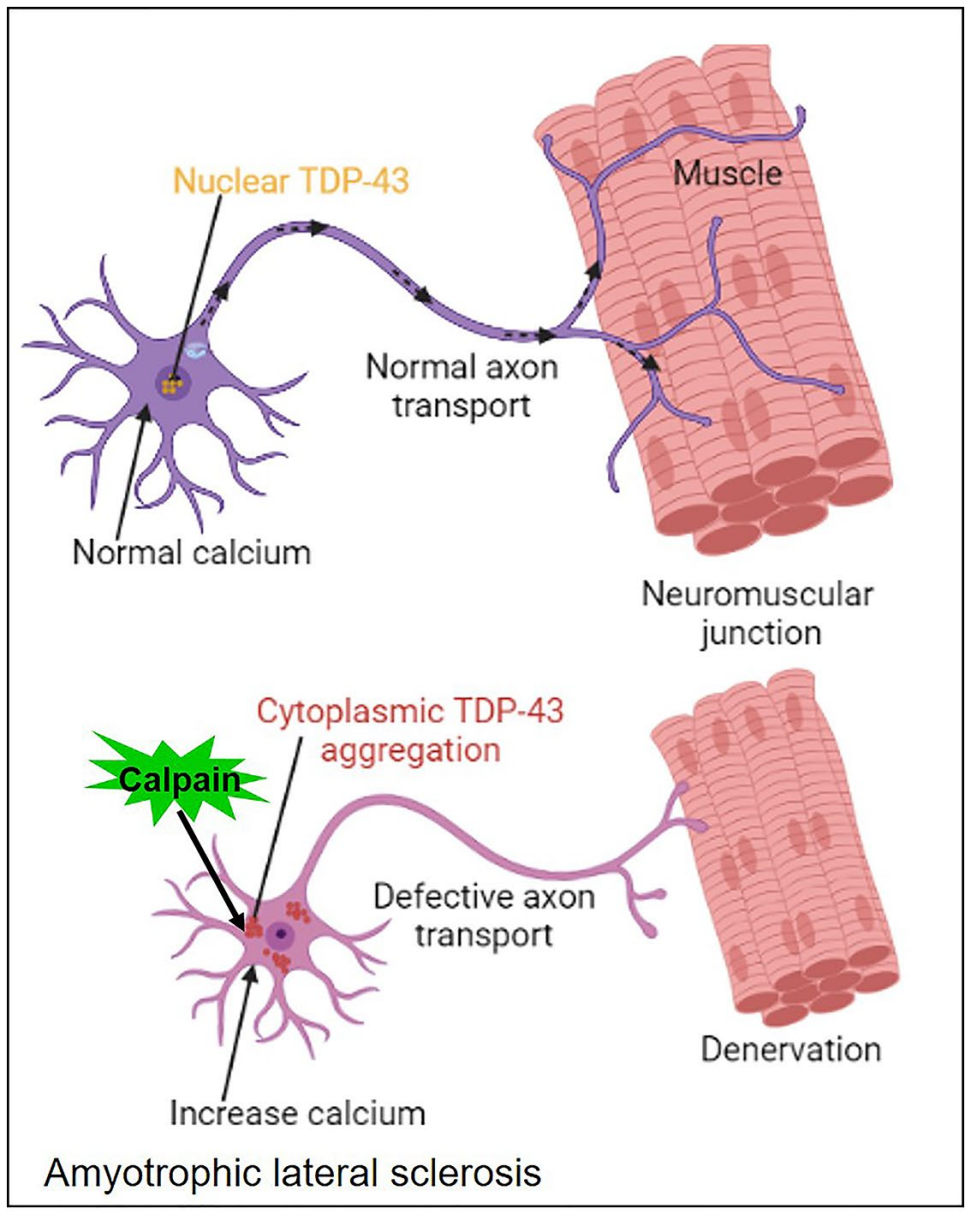
Mechanism of calpain activation in amyotrophic lateral sclerosis.

Multiple neurological disorders have been associated with calpain dysregulation (Figures 6, 7). Neurodegenerative diseases are distinguished by the gradual loss of neuronal function and structure, which results in cognitive, motor, and behavioral deficits. Calpain stands out among the proteolytic systems involved in neurodegeneration owing to its calcium-dependent activation and broad substrate selectivity (16, 83). Calpain-mediated proteolysis affects a plethora of cellular processes, including cytoskeletal dynamics, synaptic function, and apoptosis. Calpain activity dysregulation has been related to the generation of toxic aggregates and the disturbance of cellular homeostasis in a variety of neurodegenerative diseases.

AD is a progressive neurodegenerative disorder characterized by cognitive decline, memory loss, and the accumulation of amyloid-beta plaques and neurofibrillary tangles in the brain. In recent years, calpain has emerged as a critical player in the pathogenesis of AD. AD is the most common form of dementia, affecting millions of people worldwide (84). Although the precise mechanisms of AD are still unknown, mounting evidence points to calpain-mediated proteolytic processes as being particularly important in disease pathogenesis. One of the hallmarks of AD is the formation of neurofibrillary tangles, which primarily consist of hyperphosphorylated tau protein (85, 86). Calpain activation has been shown to cleave tau at multiple sites, leading to the generation of tau fragments that can self-aggregate and form tangles (Figure 6A). This calpain-mediated proteolysis disrupts the microtubule-stabilizing function of tau, leading to cytoskeletal instability and impairing axonal transport, ultimately contributing to neurodegeneration. Amyloid-beta (Aβ) peptides are derived from the proteolytic processing of amyloid precursor protein (APP). Dysregulation of APP processing can lead to the accumulation of Aβ plaques, which are neurotoxic and contribute to AD pathology (87). Calpain activation has been shown to increase the production of Aβ by promoting the cleavage of APP at specific sites, resulting in the generation of more neurotoxic Aβ fragments (88–90). In AD patients, for instance, the activation ratio of calpain-1 in the prefrontal cortex increased three times when compared to control individuals. This finding strengthens the link between calpain overactivity and neurodegenerative diseases (91). Given the significant role of calpain in AD pathology, researchers have explored the potential of calpain inhibitors as therapeutic agents. Calpain inhibitors have shown promising results in preclinical studies, reducing tau cleavage, Aβ production, and synaptic damage in animal models of AD (92). However, challenges remain in developing selective calpain inhibitors that do not interfere with the essential physiological functions of calpain.

PD is the second most common neurodegenerative disorder, affecting millions of people globally. Its hallmark features include motor symptoms, such as bradykinesia, rigidity, and resting tremors, which result from the progressive loss of dopaminergic neurons in the substantia nigra pars compacta (93, 94). While the exact cause of PD remains unknown, it is now recognized that proteolytic processes contribute significantly to neurodegeneration. Calpain has emerged as a key player in the pathological cascade of PD. α-Synuclein is a presynaptic protein implicated in PD pathogenesis (59, 95). In healthy neurons, α-Synuclein is involved in regulating synaptic vesicle dynamics and neurotransmitter release. In PD, α-Synuclein undergoes pathological misfolding and aggregation, leading to the formation of Lewy bodies, the characteristic protein clumps observed in the brains of PD patients. Calpain activation has been shown to cleave α-Synuclein into smaller toxic fragments, facilitating its aggregation and promoting Lewy body formation (96) (Figure 6B). Excitotoxicity, a process involving excessive activation of glutamate receptors, is another mechanism implicated in PD pathogenesis. Overstimulation of glutamate receptors leads to calcium influx, which in turn activates calpain. The resulting calpain-mediated proteolysis of N-methyl-D-aspartate (NMDA) receptors and other synaptic proteins disrupts normal neurotransmission, leading to neuronal damage and cell death in PD. Given the involvement of calpain in PD pathogenesis, researchers have investigated the potential of calpain inhibition as a therapeutic strategy. Preclinical studies using calpain inhibitors have shown promising results in animal models of PD, reducing α-Synuclein aggregation, mitigating mitochondrial dysfunction, and protecting dopaminergic neurons (97–99).

HD is a devastating neurodegenerative disorder that affects motor control, cognition, and behavior. The underlying cause of HD is an abnormal expansion of CAG repeats in the HTT gene, leading to the formation of mHTT protein. The aggregation of the mHTT in neurons is a central feature of HD pathology, causing neuronal dysfunction and eventual cell death (100, 101). Calpain has emerged as a critical player in the pathological cascade of HD. In HD, mHTT undergoes proteolytic cleavage, generating toxic fragments that accumulate in the cytoplasm and nucleus of neurons (102) (Figure 6C). Calpain activation has been linked to the cleavage of mHTT, releasing smaller fragments that contribute to cellular toxicity. These fragments disrupt cellular functions, interfere with proteasomal degradation, and impair mitochondrial function, ultimately leading to neuronal dysfunction and death. Advancing our knowledge of calpain in HD could pave the way for innovative therapeutic strategies to halt or slow down disease progression and improve the quality of life for HD patients (97, 102).

Amyotrophic lateral sclerosis (ALS), also known as Lou Gehrig’s disease, is a fatal neurodegenerative disorder that leads to the progressive degeneration of upper and lower motor neurons. This degeneration results in muscle weakness, atrophy, and eventual paralysis (103, 104). While the exact cause of ALS remains unknown, genetic and environmental factors contribute to disease onset and progression. Recent evidence has implicated calpain in ALS pathogenesis. Calpain activation is triggered by calcium influx into cells, and excessive calcium levels have been observed in ALS-affected motor neurons. Calpain-mediated proteolysis can lead to the cleavage of various cellular proteins, including cytoskeletal elements, ion channels, and synaptic proteins. In ALS, calpain activation has been linked to the proteolysis of critical proteins, such as neurofilaments, tau, and TAR DNA-binding protein 43 (TDP-43) (105–107). TDP-43 is a DNA/RNA-binding protein involved in RNA processing and regulation. In ALS, TDP-43 undergoes abnormal proteolysis, leading to the formation of insoluble aggregates in motor neurons. Calpain activation has been implicated in the cleavage of TDP-43, promoting its mislocalization and aggregation, which further contributes to motor neuron degeneration. Neurofilaments are major components of the neuronal cytoskeleton and are essential for maintaining axonal integrity and function (108). Dysregulated calpain activity in ALS leads to the proteolysis of neurofilaments, resulting in axonal degeneration and impaired axonal transport. This disruption contributes to the loss of motor neuron connectivity and subsequent muscle weakness.

Brain ischemia, often referred to as stroke, is a severe medical condition that occurs when the brain’s blood supply is interrupted, either due to a blocked blood vessel (ischemic stroke) or a burst blood vessel (hemorrhagic stroke) (109, 110). The lack of oxygen and nutrients leads to rapid neuronal injury and death. Calpain is activated by the calcium overload that occurs during ischemia, contributing to the cellular damage observed in stroke (88, 111). During ischemic events, calcium ions accumulate within neurons due to disrupted ion gradients. Elevated intracellular calcium levels lead to the activation of calpain, which cleaves numerous cellular proteins, triggering a cascade of molecular events that contribute to neuronal injury and cell death. Calpain activation leads to the proteolysis of cytoskeletal proteins, such as spectrin and neurofilaments, resulting in cytoskeletal disruption and destabilization of neuronal structure. This process contributes to axonal degeneration and impaired neurotransmission, further exacerbating brain ischemia’s detrimental effects (111). Ischemic stroke induces excitotoxicity, characterized by excessive release of the neurotransmitter glutamate and overactivation of glutamate receptors. This triggers calcium influx and calpain activation, leading to the cleavage of various synaptic proteins. The disruption of synaptic function and excitotoxic neuronal death further contribute to brain ischemia’s pathophysiology (112, 113).

Synaptic transmission is a fundamental process in the brain, allowing neurons to communicate and form complex neural networks. In recent years, calpains have emerged as crucial regulators of synaptic transmission in the brain (114, 115). Calpains play diverse roles in synaptic function, including modulating cellular structure, influencing transcriptional regulation, and contributing to long-term potentiation (LTP), a process that underlies learning and memory. The activation of calpain is tightly linked to changes in intracellular calcium levels. Calcium influx via NMDA receptors and calcium channels triggers calpain activation. This calcium-dependent protease then cleaves a range of downstream targets, affecting various aspects of synaptic transmission (114–116). One of the key targets of calpain in synaptic transmission is the NMDA receptor subunit NR2B. Calpain-mediated cleavage of NR2B alters the functional properties of NMDA receptors, modulating their calcium conductance and kinetics. This modification can influence synaptic plasticity and neuronal excitability, contributing to the regulation of learning and memory processes. Furthermore, calpains have been shown to cleave metabotropic glutamate receptors (mGluRs) α subunits. These receptors play critical roles in modulating synaptic transmission and synaptic plasticity (116). Calpain-mediated cleavage of mGluRs α subunits affects their functional properties, affecting neurotransmitter release and synaptic signaling (117). Moreover, calpains also target β-catenin, a protein involved in synaptic adhesion and signaling. The cleavage of β-catenin by calpain affects its stability and localization, influencing synaptic plasticity and dendritic spine dynamics (118, 119). Additionally, calpains have been implicated in regulating the abundance and function of α-amino-3-hydroxy-5-methyl-4-isoxazolepropionic acid (AMPA) receptors, which are essential for rapid excitatory synaptic transmission (120, 121). Calpain-mediated cleavage of AMPA receptors can modulate their synaptic localization and trafficking, thereby influencing the strength of excitatory synapses.

The roles of calpains in synaptic transmission regulation are complex and multifaceted. They involve a delicate balance between the proteolytic activity of calpains and the physiological functions of their target proteins. Dysregulation of calpain activity can lead to aberrant synaptic transmission and contribute to various neurological disorders, including AD, PD, and stroke (97).

## 7. Calpainopathies

**Figure 8:**
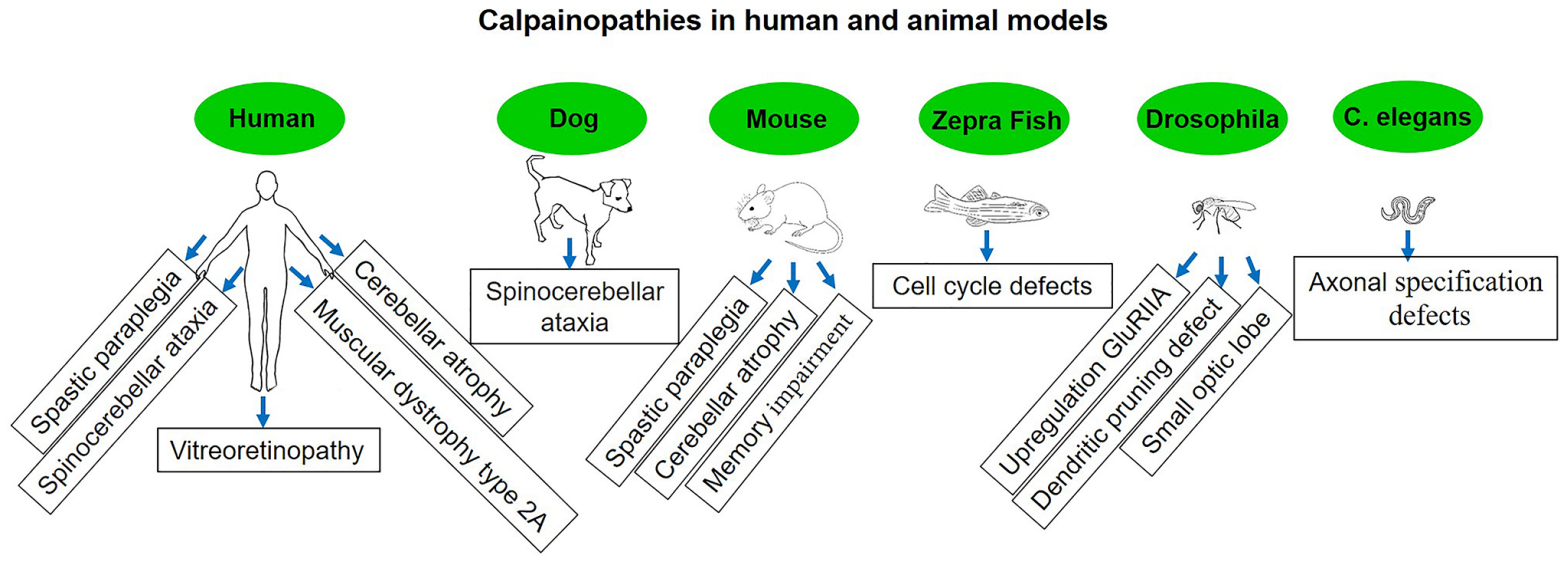
Diseases associated with calpain mutation in human and non-human models.

Gene mutations connected to the calpain enzyme system are the root cause of calpainopathies. The calpain enzyme family plays essential roles in the regulation of cellular processes, particularly those related to muscle function. It’s important to note that not all calpain mutations are associated with disease. Some mutations may not lead to any noticeable effects or may even have beneficial effects. Additionally, different mutations can result in varying degrees of severity and onset of symptoms, even within the same calpainopathy type (22) (Figure 8). The identification and understanding of specific calpain mutations are critical for accurately diagnosing calpainopathies. Genetic testing, such as DNA sequencing of the relevant calpain gene, can be performed to detect mutations and confirm the diagnosis.

Hereditary spastic paraplegia (HSP) is a group of genetically heterogeneous neurological disorders characterized by progressive weakness and spasticity (stiffness) in the lower limbs. These symptoms are a result of degeneration of the corticospinal tract, which is responsible for transmitting motor signals from the brain to the spinal cord and peripheral nerves (122, 123). There are different types of HSP, and they can be classified based on the mode of inheritance, the genes involved, and the specific symptoms observed. Mutations in the calpain-1 gene have been linked to a specific type of autosomal recessive HSP known as spastic paraplegia type 76 (SPG76). The calpain-1 gene encodes calpain-1, and mutations in this gene can lead to alterations in calpain-1 activity or function, which in turn may contribute to the development of SPG76. Mutations in the calpain-2 gene have been associated with SPG79 (124). The calpain-2 gene encodes calpain-2, and similar to SPG76, mutations in this gene can result in abnormal calpain-2 activity, potentially contributing to the development of SPG79 (124). The exact mechanisms by which calpain dysregulation leads to the neurodegenerative process in HSP are still not entirely understood. Researchers continue to investigate the molecular pathways and cellular processes influenced by calpain activity in the context of HSP to gain insights into potential therapeutic targets and treatment options.

Spinocerebellar ataxias (SCAs) are a group of hereditary, progressive neurodegenerative disorders that primarily affect the cerebellum and its connections. The cerebellum is the region of the brain responsible for coordinating movement and balance. In individuals with SCA, the function of the cerebellum is impaired, leading to difficulties with coordination, balance, and gait (125). Cerebellar atrophy is characterized by the progressive loss of neurons and shrinking of the cerebellum, the brain region responsible for coordinating movement and balance. This condition can be caused by a variety of genetic mutations, toxic exposures, or other underlying factors, leading to the dysfunction and degeneration of cerebellar neurons (126). The connection between calpain and cerebellar atrophy stems from emerging research that suggests dysregulation of calpain activity may play a role in the pathogenesis of certain cerebellar ataxias. In particular, abnormal calpain activation and proteolysis have been observed in the brains of individuals with cerebellar atrophy. Excessive calpain activity in neurons can lead to the degradation of crucial cellular components, such as ion channels, synaptic proteins, and cytoskeletal elements (127). This degradation disrupts normal cellular processes and can ultimately lead to neuronal dysfunction and death. Calpain-mediated proteolysis may exacerbate the neurodegenerative process, contributing to the loss of cerebellar neurons and consequent cerebellar atrophy. Mutations in calpain-1, for example, have been linked to spastic paraplegia (128) and SCA in dogs (129) and humans (57), with clinical symptoms often manifesting during early life or soon after maturity. Numerous investigations on calpain-related disorders have been employed on transgenic mice, *Drosophila*, zebrafish, and *Caenorhabditis elegans* (42, 43, 130, 131). These models, along with calpain activity sensors (132) that recognize activated calpain or calpain-cleaved substrates, have been regarded as important tools for revealing a clear picture in neurodegenerative diseases and other calpain-related disorders.

It is essential to recognize that the effects of calpain mutations are variable and context-dependent. For example, limb-girdle muscular dystrophy type 2A (LGMD2A) is a well-known calpain-related disease characterized by progressive muscular weakening and atrophy, primarily affecting the muscles of the limbs and pelvic girdle (133). Despite the fact that it is not typically classified as a neurodegenerative disorder because it predominantly affects the skeletal muscles as opposed to the progressive degeneration of neurons that characterizes neurodegenerative diseases, it is worth noting that they may share certain features. Some neurodegenerative disorders, for instance, may exhibit muscle paralysis due to motor neuron involvement, resulting in symptoms that are similar to those of certain forms of LGMD2A. However, the primary site of pathology and the underlying mechanisms of these conditions are distinct. LGMD2A is caused by mutations in the calpain-3 gene, which encodes the calpain-3 protein specific to skeletal muscles. The deficiency or loss of functional calpain-3 leads to the manifestation of LGMD2A as an autosomal recessive disorder with heterogeneous clinical features, including variable age of onset, delayed motor activity, and difficulty walking (134, 135). In LGMD2A, calpain-3 loses its protease function, making it important to explore potential calpain-targeted therapies that aim to restore or compensate for the loss of calpain-3 function.

Another example is autosomal dominant neovascular inflammatory vitreoretinopathy (ADNIV), a genetic disorder characterized by neovascularization and inflammation in the vitreoretinal region, leading to visual impairment. Additionally, autosomal dominant congenital cataracts associated with calpain dysfunction are characterized by opacification of the lens, resulting in visual impairment (136). Although it is not a neurodegenerative disease, it affects the retina, which may get confused with other neurodegenerative diseases affecting the retina. ADNIV is caused by mutations in the calpain-5 gene, which encodes the calpain-5 protein, have been linked to autosomal dominant posterior polar cataracts. Calpain-5 is expressed in various tissues, with a prominent presence in the central nervous system (137).

Research in the field of genetics and calpain-related disorders continue to advance, offering hope for improved diagnosis, management, and potential therapeutic interventions for individuals affected by these conditions. Understanding the genetic basis of calpain mutations contributes to a deeper comprehension of these disorders and paves the way for targeted treatments in the future.

## 8. Concluding remarks and future implications

Recent research highlights that calpains play a critical role in neurodegeneration, demonstrating their significance not only in cellular damage but also in the development and remodeling of neurons. There has been evidence of calpain overexpression in a number of systemic diseases and neurological disorders. Although substantial progress has been made in understanding the role of calpains in brain activity and pathological disorders, additional research is necessary to overcome the obstacles involved with using calpain inhibitors as a therapeutic approach. It is critical to get a better understanding of the signaling cascades regulated by calpain activation under various physiological scenarios. It becomes critical to understand the regulatory processes involved in the activation and inhibition of calpain.

Calpains have a vital function in the process of neuronal remodeling and are closely linked to neurodegeneration. Disruptions in neuronal remodeling are known to contribute to the development of neuropsychiatric disorders, including autism and schizophrenia (46, 138). Gaining a deeper understanding of how neurites undergo remodeling can offer valuable insights into the underlying pathophysiology of related mental diseases. While the use of calpain inhibitors for the treatment of these disorders may seem promising, it poses challenges due to the potential dual effects of non-specific calpain inhibitors. To address this issue, several approaches can be explored. Advancements in imaging techniques, such as fluorescent probes and genetically encoded sensors, have enabled real-time visualization and quantification of calpain activity in living cells and tissues. These technological advancements, combined with high-throughput screening approaches, provide powerful tools for studying calpain biology and identifying potential drug candidates. Calpain has emerged as a key player in various physiological and pathological processes. Understanding the complex regulatory mechanisms and molecular interactions of calpain is crucial for unraveling its role in diseases and developing effective therapeutic strategies. Future research should focus on elucidating the structural and functional aspects of calpain, identifying novel calpain substrates, and translating these findings into clinical applications for improved patient care and management. In conclusion, the multifaceted functions of calpain make it an exciting area of research with significant implications for biomedical advancements.

It’s important to note that while calpain inhibitors and neuroprotective strategies show promise in preclinical studies, their translation to clinical use in humans is complex and challenging. Neurodegenerative diseases are multifactorial, and targeting a single pathway like calpain may not be sufficient to halt disease progression entirely. As such, comprehensive therapeutic approaches targeting multiple pathological mechanisms are likely to be the most effective in treating neurodegenerative disorders. Clinical trials are ongoing to assess the safety and efficacy of calpain-based therapeutic interventions, and further research is required to fully understand the potential of calpain signaling as a target in neurodegenerative diseases.

## Author contributions

EM, HA-A, and MFA contributed to the conception and design of the study. EM and AMA wrote the first draft of the manuscript. TH, GM, HA-A, and MFA wrote sections of the manuscript. All authors contributed to the article and approved the submitted version.

## Conflict of interest

The authors declare that the research was conducted in the absence of any commercial or financial relationships that could be construed as a potential conflict of interest.

## Publisher’s note

All claims expressed in this article are solely those of the authors and do not necessarily represent those of their affiliated organizations, or those of the publisher, the editors and the reviewers. Any product that may be evaluated in this article, or claim that may be made by its manufacturer, is not guaranteed or endorsed by the publisher.
